# New evidence on the rural poverty and energy choice relationship

**DOI:** 10.1038/s41598-023-29285-6

**Published:** 2023-02-27

**Authors:** Salvatore Di Falco, Gary Lynam

**Affiliations:** 1 Institute of Economics and Econometrics, Geneva School of Economics and Management, University of Geneva, Geneva, Switzerland; 2 Institute for Environmental Studies, University of Geneva, Geneva, Switzerland

**Keywords:** Energy and society, Environmental economics, Sustainability

## Abstract

We combine a global micro level dataset that includes 17 different rural Sub-Saharan countries with satellite information about precipitation during the growing season to estimate the impact of economic conditions on energy choice. Differently from the existing literature, we aim to causally estimate the impact of household welfare variation on the likelihood of choosing a specific energy source. It is found, consistent with theory, that increases in income do determine an increase in the likelihood of using relatively cleaner and more efficient sources of fuel. We find, however, that this impact is quantitatively very small. Results hold conditional on assets, wealth and a large battery of controls and fixed effects. Policy implications are developed.

## Introduction

Around 3 billion people in the world are “energy poor”, meaning they rely upon harmful fuel sources like biomass-generated fire for their cooking and heating. The reduction of the pervasive use of wood-based combustibles implies two particularly important environmental and health objectives. First, less pressure on forests for the provision of energy sources. Second, less detrimental effects of indoor pollution as the use of these fuel sources have negative impacts on health through the inhalation of fine particles. These particles result in over 2.8 million premature deaths worldwide^1,2^. Transition to clean and affordable energy sources is a fundamental objective of sustainable development (Sustainable Development Goal no 7).

Economic forces can play an important role in facilitating energy choice and potential transition. A common conjecture is that the choice of a more efficient and clean energy source is a direct, or at least a natural, consequence of an increase in living standards. Energy source choices, as they reflect different levels of environmental quality, can be considered a luxury good. Increases in income, among other things, implies greater control over resources and more awareness of the effect of energy choice^3,4^. This conjecture is behind the widely studied energy ladder hypothesis^5–10^. In the developing and emerging world that means going from traditional inefficient sources (fuel wood, charcoal, kerosene etc.) to more efficient modern sources (liquid gas, electricity), once higher levels of income are attained. This paper tests this hypothesis by presenting evidence from households in 17 rural Sub-Saharan countries. Differently from the existing literature using multi-country datasets, our aim is to causally estimate the impact of income changes on energy choice^11–14^. To this end we use random variation in rainfall during the growing season of the main harvested crops. In rural environments, rainfall variations during this period provide a clean source of variation (also called natural experiment^15^) that is not systematically related to other variables. For instance, households experiencing less than average rainfall will obtain lower harvests and thus income. Using random variation in rainfall during the growing season, therefore allows us to make causal inference and circumvent the critical issue of accurate income measurement in developing countries. Our study focuses on such an environment, as we only use observations of rural farm households in the Demographic Health Survey (DHS). Table 1 shows that 80% of the sample relies on rural assets such as agricultural land for their living. Given the very low levels of irrigation, the welfare of these farm households heavily depends on the rainfall they obtain during the growing season.

**Table 1:** Variables definition and descriptive statistics.

Variable name	Description	Mean	Sd	Min	Max
Household owns farmland	1—owns farmland, 0—otherwise	0.8		0	1
Household owns livestock	1—owns livestock, 0—otherwise	0.7		0	1
# Household members	Count of full-time members in household	5.07	3.16	1	66
Household head age	The age of the household head	45.62	16.49	11	97
Household head education	Highest achievement of education of household head: none, (in)complete primary, (in)complete secondary and tertiary	3.88	4.39	0	24
Wealth index	The wealth index is calculated using data on a household’s ownership of selected assets, such as electrical appliances; materials used for housing construction; and types of water access and sanitation facilities. Generated with a statistical procedure known as principal components analysis, the wealth index places individual households on a continuous scale of relative wealth. DHS separates all interviewed households into five wealth quintiles				
Precipitation seasonal mean	The wet seasonal precipitation-mean of the cluster (100 mm)—derived from the Climate Hazards Group InfraRed Precipitation with Station data (CHIRPS)^16^	10.40	6.43	0.009	42.82
Precipitation seasonal anomaly	The cluster standard deviation of the wet seasonal precipitation-mean from the 20-year seasonal precipitation mean (1997–2017)	− 0.15	1.05	− 3.57	3.03
Precipitation seasonal anomaly category type	Taking − 0.5 to 0.5 standard deviations (sd) to be the normal precipitation seasonal anomaly, four categories are defined as follows: Extreme drought (more than − 2 sd from the 20-year mean) Severe drought (between − 2 and − 0.5 sd from the 20-year mean) Severe flooding (between 0.5 and 2 sd from the 20-year mean) Extreme flooding (more than 2 sd from the 20-year mean)				
Year temperature mean	The yearly temperature mean of the cluster (Celsius)—derived from the MOD11A1 V6 satellite^17^	30.77	4.060	17.36	49.47
Soil moisture	The soil moisture (in the layer of 0–100 cm depth) mean of the cluster (kg/m^2^)—derived from the GLDAS-2 satellite using the 4-soil layer Noah model^18^. This is a measure of the quantity of water moisture in kilograms per square metre of a depth of 100 cm	210.3	55.37	53.27	400.3
Country electricity access (% of pop)	The percentage of the country’s population that has access to the electricity grid, as given by The World Bank database—accessed June 2019				

We then use a multinomial logit to estimate the causal impact of random household income variation on the likelihood of moving up the energy ladder. To further address omitted variables concerns, a large battery of controls is added. These include household’s assets, wealth, education, and other socio-economic characteristics of the household head as well as access to electricity and infrastructure. A set of time and geographic fixed effects is also included to control for sources of unobservable heterogeneity that are space and time invariant.

## Data sources and variables

The dataset was generated by combining USAID’s Demographic Health Survey (DHS) country level household surveys with publicly available satellite information on precipitation accessed through the Google Earth Engine tool. The DHS survey information contains a set of data from representative national household surveys. Several years between 2006 and 2016 are available for each country, resulting in a pooled cross-section of 460,780 household observations (see Table A1). Filtering for rural households (defined as households residing in the countryside, as opposed to urban households that live in cities and towns), we are left with 310,707 observations across 17 sub-Saharan Africa countries: Burkina Faso, Burundi, Ethiopia, Ghana, Kenya, Lesotho, Liberia, Madagascar, Mali, Malawi, Mozambique, Namibia, Nigeria, Rwanda, Senegal, Sierra Leone, and Zimbabwe. The survey records the primary source of energy used for cooking by the household in each round. These are: biomass (e.g., crop residue, dung, fuel wood), charcoal, kerosene, liquid petroleum gas (LPG), and electricity. Each source represents a rung in the energy ladder, from the least efficient and most detrimental to household health and the environment, to the most efficient and clean. To build our key dependent variable, we organize and categorize these fuels into three distinct rungs of the ladder (as in Van der Kroon et al.^10^). The first rung includes the traditional fuel sources (biomass), the second includes the transitional sources (charcoal and kerosene), and the third encompasses the modern sources such as LPG and electricity. The DHS survey also records a large set of relevant controls. These include household assets (if the household owns any land or livestock), household wealth, the number of household members, the age of the household head, and the household head’s level of education. We expand the list of controls by including two important variables at a higher level of aggregation. First, we include a control for the extent of access to electricity at the country level (the percentage of population with electricity access). Second, we include a control for soil quality as this capture the level of natural capital available to the household.

As previously mentioned, we use random variation in rainfall during the growing season as a measure of income variation at the household level. The GPS points of household clusters were used in conjunction with the Google Earth Engine tool to calculate relevant precipitation data for the corresponding year of the country survey. All the geographic data extractions used these publicly available cluster locations provided by the DHS Program surveyors. The GPS location of each cluster centre is recorded during either the fieldwork or listing stage of the survey process. Geo-masking methods are then used to ensure the confidentiality of the DHS respondents, displacing the cluster centres from their true locations by up to 10 kms. Therefore, we take this distance as the diameter of the circular area we calculate our geospatial data in, with the cluster GPS point as its centre. All geospatial data are calculated as annual means and are lagged 1 year prior to the DHS survey interview dates. The one exception for this is the main variable of interest, precipitation, which is calculated across the average planting/sowing month to the average harvest month for the main staple crops (cereals) in each country. The cereal crop calendar is presented in the Appendix.

We obtained the amount of rainfall (mm during the growing season) and calculated a set of precipitation anomaly dummy variables. We first computed a precipitation anomaly as the standardized difference between the observed level of rainfall during the growing season minus its long-term average (20-year seasonal precipitation average). This anomaly variable thus allows us to measure how much (in terms of standard deviation) the rainfall during the growing season is different from the long-term seasonal mean (20 years). These values were then converted into 4 categories: extreme drought (less than 2 standard deviations), mild drought (between less than 2 standard deviations and 0.5), mild to severe flooding (more than 0.5 standard and less than 2), extreme flooding (torrential wet season with more than 2 standard deviations). This approach allowed us to analyse the seasonal extremes which let us investigate if there are any negative effects on the primary fuel choice. The definitions of the variables used in the analysis and the summary statistics are reported in the Table 1.

## Model

In order to model the household primary fuel choices, several multinomial logit (MNL) regressions were carried out^19^. MNL is a standard technique used in quantitative studies on energy transition which assumes households have a preference of energy type that maximises their perceived indirect utility function (more details are provided in A3). This allows one to assess how a given driver (income in this study) affects the choice of energy for a particular use conditional on a set of controls such as household head age, education, number of household members and so on^11^. The dependent variable is defined over three fuel type categories for their main source of cooking fuel, i.e. traditional, transitional and modern. For the ith household faced with the jth choice at time *t*, the utility function can be written as:

$${U}_{ijt}={\beta }^{^{\prime}}{X}_{ijt}+ {\varepsilon }_{ijt}$$

where, ${X}_{ijt}$ is a vector of explanatory variables for household *i*, in the region/country *j* at time *t*. $\beta$ is a vector of unknown regression coefficients and ${\varepsilon }_{ijt}$ is the error term. More details on the method are reported in the Appendix. For simplicity of exposition, we let the matrix *X* represent the key rainfall variable, the controls, and the fixed effects.

## Results

Table 2 reports the estimation results. The omitted baseline is the transitional category. Column (1) and (3) report the estimated marginal effects for the precipitation anomalies without controls. Columns (2) and (4) reports the marginal effects once all the controls are included. All specifications include time and country fixed effects (an alternative specification with regional fixed effects was excluded due to asymmetry in the variance matrix). Results are very consistent across specifications. Extreme drought and flooding have a positive impact on the probability that the household will utilise traditional biomass-based energy sources. Coherently with the energy ladder hypotheses, the same extreme variables have a negative impact on the probability to use modern energy sources. However, it should be noted that the magnitude of this effect is very small. Negative income shocks, such as extreme drought or flooding, increases the probability of going from transitional to traditional sources by less than 1%. Similarly, the same negative variation in income reduces the probability of choosing more modern energy sources by less than 1%. While the estimated income variations are associated with movement up or down the energy ladder consistent with the prior, the changes in probability seem very small. The results are robust to the inclusion of a large battery of controls. These include owning either farmland or livestock (which acts as a proxy for agricultural assets), soil moisture and temperature (which act as controls for agricultural production), a wealth index, education, and household size. Results are also robust to the inclusion of controls for access to electricity and vegetation conditions. We avoid making any interpretation of the controls’ coefficient estimates as these may pick up simple correlations.

**Table 2:** Multinomial logits outcomes. Marginal effects.

Variables	(1)	(2)	(3)	(4)
	Trad base	Trad control	Mod base	Mod control
Extreme drought	0.0186*** (0.00475)	0.00565*** (0.00132)	− 0.000616 (0.000827)	− 2.22e−05** (1.13e−05)
Mild wet season drought	0.0107*** (0.00349)	− 0.000368 (0.000980)	− 0.00309*** (0.000695)	− 1.58e−05 (9.86e−06)
Mild to severe flooding	− 0.000185 (0.00397)	0.00160* (0.000952)	0.000560 (0.000869)	− 2.10e−05* (1.14e−05)
Extreme flooding	0.0177*** (0.00620)	0.00558*** (0.00179)	− 0.000835 (0.00101)	− 3.21e−05* (1.67e−05)
Controls	No	Yes	No	Yes
Country + time fixed effects	Yes	Yes	Yes	Yes
Observations	304,010	263,349	304,010	263,349

Clustered standard errors in parentheses. ***p < 0.01, **p < 0.05, *p < 0.1.

As further robustness checks, we use alternative metrics for the precipitation during the growing season. First, we use precipitation mean and its quadratic term. Second, we use the log of the precipitation mean. Both parametric specifications allow to further test the hypotheses consider the potential non-linear nature of the relationship between income and fuel choices. Results are again consistent and are reported in the Tables 3 and 4.

**Table 3:** Model estimated replacing drought indicator with seasonal rainfall.

Variables	(1)	(2)	(3)	(4)
	Trad base	Trad control	Mod base	Mod control
Precipitation seasonal mean (100 mm)	0.000927 (0.00075)	0.00134*** (0.00028)	− 0.000743*** (0.00014)	− 1.37e−05*** (2.95e−06)
Precipitation seasonal mean quadratic	− 0.00013*** (2.42e−05)	− 4.73e−05*** (8.14e−06)	3.08e−05*** (4.60e−06)	3.92e−07*** (9.44e−08)
Controls	No	Yes	No	Yes
Country + time fixed effects	Yes	Yes	Yes	Yes
Observations	304,010	263,349	304,010	263,349

Clustered standard errors in parentheses ***p < 0.01, **p < 0.05, *p < 0.1.

**Table 4:** Model estimated replacing drought indicator with natural log of precipitation.

Variables	(1)	(2)	(3)	(4)
	Trad base	Trad control	Mod base	Mod control
Log of precipitation seasonal mean	− 0.0243*** (0.00313)	0.000719 (0.00110)	− 0.00286*** (0.000389)	− 4.65e−05*** (6.63e−06)
Controls	No	Yes	No	Yes
Country + time fixed effects	Yes	Yes	Yes	Yes
Observations	304,010	263,349	304,010	263,349

Clustered standard errors in parentheses ***p < 0.01, **p < 0.05, *p < 0.1.

## Conclusions

This paper contributes to the energy transition literature by revisiting the energy ladder hypothesis. We expand on the existing literature in two dimensions. First, by analysing a large micro dataset that encompasses 17 countries. Second, by estimating the causal effect of rural income variations on fuel choice. The results show a weak impact of extreme precipitations on the choices of energy, only increasing the probability of choosing more traditional sources by approximately 1%. Given that precipitation captures the income variations derived from agricultural output, we can observe that the income channel predicted in the energy ladder theory is weak relative to the expected impact. These results highlight that increases in economic conditions need to go beyond economic growth to reach the Sustainable Development Goal 7 of achieving universal access to clean energy.

It should be stressed that our results are consistent with the energy stacking literature^20,21^, where household characteristics (such as education, available labour for collecting firewood and cultural preferences), as well as conditions external to households (local market prices and stable supplies, as well as national policy programmes/economic performance) are more important factors in determining energy choices among rural households^10^. This literature also states that households do not arbitrarily switch from one fuel to another for all household needs, but instead stack energy sources for use in different tasks. As several studies support that households indeed stack their stove fuel choices, this implies policy interventions should target the adaption of the cleanest stack and consider the local context for successful and long-lasting transition, rather than promoting the use of single fuel-stove combinations that do not meet the targeted community’s needs^22,23^. However, to explore this further and thus better inform policy, national survey data needs to start focusing beyond primary fuel and look at energy stacks for household energy needs.

## Supplementary Information

Supplementary Information.

## Data Availability

The datasets used and analysed during the current study available from the corresponding author on reasonable request.
