# Supplementary material for: New evidence on the rural poverty and energy choice relationship
Source: Sci Rep. 2023 Feb 27;13:3320. doi: 10.1038/s41598-023-29285-6 (PMC9971361; doi:10.1038/s41598-023-29285-6)
Supplement: Supplementary file 1 — Supplementary Information. [file 41598_2023_29285_MOESM1_ESM.pdf]

Supplementary Material

A1. Country years used in survey

**Burkina Faso**	2010, 2014	**Malawi**	2010, 2012, 2014
**Burundi**	2010, 2012	**Mozambique**	2011, 2015
**Ethiopia**	2011, 2016	**Nambia**	2006, 2013
**Ghana**	2008, 2014, 2016	**Nigeria**	2008,2013
**Kenya**	2008, 2014	**Rwanada**	2010, 2014
**Lesotho**	2009, 2013	**Senegal**	2010, 2014, 2016
**Liberia**	2011, 2013	**Sierra Leone**	2008, 2013, 2016
**Madagascar**	2008, 2016	**Zimbabwe**	2005, 2010, 2015
**Mali**	2012, 2015		

A2. Country Planting and harvest months (1 to 12) for cereals (FAO)

**Country**	**Planting month main season**	**Harvest month main season**	**Planting month second season**	**Harvest month second season**	**Rainy months**
Burkina Faso	6	10			5
Burundi	2	5			4
Ethiopia	4	10			7
Ghana	3	10			8
Kenya	3	6	10	12	7
Lesotho	10	3			6

Liberia	1	12	12
Madagascar	11	4	6
Mali	7	9	3
Malawi	11	4	6
Mozambique	11	4	6
Nambia	12	3	8
Nigeria	4	10	7
Rwanda	9	5	3
Senegal	6	10	5
Sierra Leone	4	11	8
Zimbabwe	11	3	5

A3: Details on the model

If the household makes a choice j, we then assume that U_{ij} is the maximum among the three fuel types, i.e. j is chosen if $U(fuel\ j) > U(fuel\ k)$ where j ≠ k. The observed fuel choice is defined as a vector $Y_i = Y_{ij}$ of three dummy variables taking value 1 if the household's choice is the jth alternative, and value 0 otherwise (consider j takes the following values: 0 - traditional, 1 - transitional, 2 - modern). If we consider the choice probabilities at the mean of every variable in X, we have:

$$Prob(Y_{ij} = j) = \frac{\exp\,(\beta_j' \bar{x_i})}{\sum_{m=1}^{j} \exp\,(\beta_m' \bar{x_i})}, \qquad j = \{0,1,2\}$$

Where m = base outcome choice of fuel. We take transitional fuel use as the base outcome as it allows the interpretation from a mid-point on the likelihood of using traditional or modern fuel

sources in lieu of transitional ones. The marginal effects are taken at the mean of all explanatory variables for each precipitation variable model. Given this approach, four columns are presented with the first two representing the marginal effects from the mean on choosing traditional fuels over transitional and the last two representing the choice of modern sources. In each pair of outcomes, the first column excludes controls (but keeping country and time fixed effects), while the adjacent columns respectively show the results includes such controls.

A4: Cluster buffer methodology

For the purposes of this study, a 10-kilometre diameter circular buffer was created around each point to calculate satellite indicator statistics. This was done to be sure the original cluster centre would be included and given the relative homogeneity over such a small area. Depending on the indicator, either the sum or mean of the year the survey took place is taken for each pixel within the country (1 pixel = 1km^2). The mean and standard deviation (as well as maximum and minimum values in some cases) were then taken over the cluster buffer area. All derived data set cell sizes are at ~1000 m.
